# Dormant cancer cells and polyploid giant cancer cells: The roots of cancer recurrence and metastasis

**DOI:** 10.1002/ctm2.1567

**Published:** 2024-02-16

**Authors:** Yuqi Jiao, Yongjun Yu, Minying Zheng, Man Yan, Jiangping Wang, Yue Zhang, Shiwu Zhang

**Affiliations:** ^1^ School of Integrative Medicine Tianjin University of Traditional Chinese Medicine Tianjin China; ^2^ Department of Pathology Tianjin Union Medical Center Tianjin China; ^3^ Department of Pathology Tianjin Union Medical Center Nankai University Tianjin China

**Keywords:** cell cycle arrest, dormancy, endoreduplication, polyploid giant cancer cells

## Abstract

Tumour cell dormancy is critical for metastasis and resistance to chemoradiotherapy. Polyploid giant cancer cells (PGCCs) with giant or multiple nuclei and high DNA content have the properties of cancer stem cell and single PGCCs can individually generate tumours in immunodeficient mice. PGCCs represent a dormant form of cancer cells that survive harsh tumour conditions and contribute to tumour recurrence. Hypoxic mimics, chemotherapeutics, radiation and cytotoxic traditional Chinese medicines can induce PGCCs formation through endoreduplication and/or cell fusion. After incubation, dormant PGCCs can recover from the treatment and produce daughter cells with strong proliferative, migratory and invasive abilities via asymmetric cell division. Additionally, PGCCs can resist hypoxia or chemical stress and have a distinct protein signature that involves chromatin remodelling and cell cycle regulation. Dormant PGCCs form the cellular basis for therapeutic resistance, metastatic cascade and disease recurrence. This review summarises regulatory mechanisms governing dormant cancer cells entry and exit of dormancy, which may be used by PGCCs, and potential therapeutic strategies for targeting PGCCs.

## Graphical abstract

1. PGCCs have emerged as a key regulator of tumour dormancy and reactivation.

2. Abnormal cell cycle regulation in PGCCs enables them to enter a reversibly dormant state and evade cancer therapy.

3. PGCCs achieve dormancy through endoreduplication and cell cycle arrest.

4. PGCCs can exit dormancy and recur via asymmetric division, thereby generating aggressive diploid progeny.

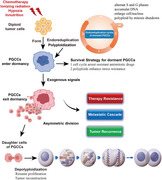

## 1 INTRODUCTION

Cancer dormancy is closely associated with metastasis. In this work ‘The Spread of Tumors in the Human Body’, Rupert A. Willis was the first to use the term, dormancy, to refer to cells that disseminate but are in growth arrest.^1^ Later, Hadfield defined the dormant cancer cell as ‘malignant cells, although remaining alive for relatively long periods, show no evidence of proliferation during this time, yet retain all their former and vigorous capacity to proliferate’.^2^ Dormant cancer cells are characterised by cell cycle arrest, indicating that they do not actively divide or proliferate.^3^, ^4^, ^5^ Dormant cancer cells are usually in the non‐proliferative G0 or G1 phases. This state of cell cycle arrest allows dormant cancer cells to evade detection and destruction by the immune system, and traditional cancer treatment.^6^ However, under certain conditions (such as changes in the microenvironment or immune system), dormant cells can reactivate and proliferate, leading to metastasis and recurrence.^7^, ^8^, ^9^

Polyploid giant cancer cells (PGCCs) contribute to tumour heterogeneity and play essential roles in tumour progression.^10^ The number of PGCCs is relevant to tumour grade, lymph node metastasis, recurrence, chemoresistance and poor prognosis.^11^ PGCCs are also known as polyploid aneuploid cancer cells (PACC),^12^ multinucleated giant cancer cells,^13^ osteoclast‐like giant cells and cancer‐associated macrophage‐like cells.^14^ PGCCs have either a single giant or multiple nuclei. Multinuclear PGCCs are more likely to occur when nuclear replication and separation is complete, and cytoplasmic separation fails. PGCCs with single giant nuclei appear when nuclear and cytoplasmic separation fail simultaneously.^15^, ^16^, ^17^ PGCCs usually develop and enter a dormant state in response to stress, such as chemotherapy, radiation and hypoxia microenvironment.^11^, ^18^, ^19^, ^20^, ^21^, ^22^, ^23^, ^24^ The features of dormant PGCCs are abnormal cell cycle‐related protein expression and proliferative arrest,^25^ resulting in an increase to the number of unbalanced chromosomal copies.^26^, ^27^ Since dormant PGCCs are usually in a non‐proliferative state, the toxic effects of systemic therapies are evaded and residual PGCCs later reactivate and proliferate, leading to tumour recurrence.^19^ PGCCs are the driving force behind tumour recurrence and are a new focus in cancer research. Understanding the dormant characteristics of PGCCs and the biological mechanisms underlying reactivation are crucial to prevent metastatic growth and recurrence. In this review, we introduce the molecular mechanisms behind cancer cell entry and exit from dormancy and comprehensively describe why PGCCs are dormant. In addition, the abnormal cell cycle and pro‐cancer mechanisms of PGCCs after reactivation from dormancy are explored. We also discuss the potential treatment strategies for eliminating PGCCs.

## 2 DORMANCY AND CANCER

Tumour cell dormancy is an important stage of tumour metastasis.^28^, ^29^, ^30^ Highly motile cell populations exist in early lesions and can enter dormancy at distant sites.^31^, ^32^ To maintain dormancy, tumour cells suppress genes required for DNA synthesis or cell division and promote expression of anti‐apoptotic, anti‐aging and anti‐differentiation genes.^33^, ^34^ Dormant cancer cells share several identical characteristics and signalling pathways with cancer stem cells, including recurrence, the ability to metastasize and evade the immune system.^35^, ^36^, ^37^, ^38^, ^39^ Dormancy is defined by two important cellular characteristics: (1) growth arrest and (2) dissemination, which provide cancer cells the ability to leave the primary tumour.^1^ Growth arrest allows dormant cancer cells to retain their proliferative capacity, which can be reversed by a series of intrinsic and cell‐extrinsic factors.^40^ Proliferation occurs only after dormancy is disrupted, leading to the formation of tumours.^41^

Growth arrest can allow disseminated cancer cells (DCCs) undergo different cellular states (migration, quiescence and proliferation)^42^, ^43^, ^44^ and evade anticancer therapy and immune surveillance.^45^, ^46^ During dissemination, DCCs must overcome physical barriers by degrading the extracellular matrix (ECM) to invade the matrix, migrating along the ECM to leave the tumour and undergoing intravasation then extravasation by degrading the vascular basement membrane.^47^ After the initiation of migration, DCCs reach various niches and actively settle into the new organ environment to establish dormancy.^48^, ^49^, ^50^ After signals arising from the microenvironment, DCCs can switch from their dormant state and reemerge to proliferative growth.^8^ Epithelial–mesenchymal transition (EMT) is crucial gene expression program associated with cell quiescence and metastasis.^51^, ^52^, ^53^, ^54^ EMT is a key mechanism for initiating cell migration and maintaining the dormancy phenotype by utilizing mesenchymal programs that promote invasiveness and reduce proliferation. In contrast, epithelial programs reactivate cells from dormancy and induce metastatic growth.^46^ Cytokines such as transforming growth factor‐β (TGF‐β),^55^ interferon‐γ^56^ and interleukin‐8 (IL‐8)^57^ help maintain cell quiescence at distant metastatic sites.

## 3 MOLECULAR MECHANISMS OF CANCER CELL ENTRY AND EXIT FROM DORMANCY

Regulatory mechanisms in dormant cancer cells can be divided into two categories: intracellular and extracellular. Intracellular regulatory mechanisms are influenced by cell cycle arrest,^58^ redox signalling,^59^ metabolic reprogramming^60^ and autophagy.^61^ Extracellular regulatory mechanisms are influenced by factors such as the microenvironment^62^ and immune system.^63^ For example, intracellular factors including CD133, Sox‐2 and E‐cadherin are used to assess the dormancy state of colorectal cancer cells in the liver, whereas vimentin, Ki‐67, c‐Myc, cyclin D1 and vascular endothelial growth factor (VEGF) are used to assess the cell‐awakening state.^64^ Regulation of the dormant cancer cell cycle involves multiple factors and pathways, such as cyclin‐dependent kinase (CDK) inhibitors, which inhibit CDK activity and promote quiescence by preventing retinoblastoma protein (Rb) phosphorylation, which is required for G1/S transition.^65^ P53 and cell cycle‐related proteins, TGF‐β signalling and p38/mitogen‐activated protein kinases (MAPK) signalling pathways can also promote quiescence by inhibiting CDK/cyclin and regulating cell growth, survival and differentiation. Many transcription factors, including P53,^66^ Foxo family^67^ and Myc,^68^, ^69^ nuclear receptor subfamily 2, group F, member 1^70^ are involved in the regulation of quiescence and dormancy. Extracellular factors mediates dormancy including angiogenic (such as VEGF,^71^ Angiostatin^72^), immune (such as IL‐3,^73^ interferon‐γ,^74^ growth arrest‐specific protein 6,^75^ leukaemia inhibitory factor receptor^76^), ECM (such as TGF‐β1,^77^ TGF‐β2,^35^ insulin‐like growth factor 1,^78^ E‐selectin,^79^ bone morphogenetic protein 7 (BMP7)^80^) and stress‐induced factors (such as p38 MAPK^81^). Cancer cell quiescence is a self‐protective mechanism that prevents destruction from hypoxia, nutrient deprivation, immune surveillance and chemotherapy.^82^

### 3.1 P53 and cell cycle‐related proteins are involved in the dormancy of cancer cells

P53 and cell cycle‐related proteins are primary markers of cancer cell dormancy.^83^, ^84^ P53 activity in quiescent cells is higher than it in proliferating cells.^85^, ^86^ High expression of P53 regulates the activation of ubiquitin ligase anaphase‐promoting complex responding to 5‐fluorouracil‐induced dormancy in non‐small cell lung cancer.^52^ P53 expression inhibits the CDK/cyclin complex, blocking cell cycle transition from the G1/S or G2/M phase.^87^ CDK support the maintenance and escape of dormancy. Signals of dormancy typically increase the expression levels of CDK inhibitors or phosphorylation of Rb.^45^, ^88^ Various growth factors and signalling pathways can affect CDK activity and the proliferation/quiescence outcomes of cancer cells.^65^ Ki67 and the CDK inhibitor (p27) are often used as markers of dormant tumour cells.^69^ In gallbladder cancer (GBC), the expression of F‐box/WD repeat‐containing protein 7 and p27 is up‐regulated and associates with reduced GBC cell proliferation and entry into dormancy.^89^ Moreover, CDK4 is down‐regulated and DCCs is dormant in head and neck squamous cell carcinoma.^90^ P53 and cell cycle‐related proteins are key factor regulating dormant cancer cells via influencing cell cycle.

### 3.2 TGF‐β signalling pathway is involved in the dormancy of cancer cells

TGF‐β signalling with various cellular functions are essential in tumour dormancy. TGF‐β signalling can disrupt persistent proliferation signals in cancer cells and maintain the quiescent state of dormant cells.^91^, ^92^ TGF‐β and other growth inhibitory signals can suppress the proliferation of DCCs.^93^, ^94^ Reactive oxygen species (ROS) stimulate TGF‐β2 on tumour cell membranes and activate p38, leading to cancer cell dormancy.^95^ In addition, TGF‐β2 signalling, Rho‐Associated Coiled‐Coil Kinase (ROCK), α5β1 integrin (mainly distributed in fibroblasts, and its ligand is fibronectin) and αvβ3 integrin supports cancer cell of dormancy. Matrix metalloproteinase 2 release reactivates dormant cancer cells to proliferate.^96^ In the lung, TGF‐β promotes niche settlement by inducing angiopoietin‐like 4 (a pro‐inflammatory factor), a mediator that helps cancer cells penetrate tissue.^97^ TGF‐β2 combined with TGF‐β‐receptor‐I, TGF‐β‐receptor‐III and SMAD1/5 activation can induce the dormancy of lung metastatic foci of head and neck squamous cell carcinoma.^90^ The activity of TGF‐β signalling plays a critical role in the dormancy of lung metastatic cells by down‐regulating stimulators of interferon genes and C motif chemokine 5 (CCL5) expression.^98^ TGF‐β signalling play a pivotal role in cellular dormancy.

### 3.3 p38/MAPK signalling pathway is relevant to the dormancy of cancer cells

As a tumour suppressor, p38/MAPK promotes cancer dormancy.^81^, ^99^ For example, Regucalcin can promote the dormancy of cancer cells by inducing p38 MAPK activation and forkhead box M1 (FOXM1) inhibition.^100^ Glypican‐3 can induce tumour dormancy through the activation of p38 MAPK signalling pathway.^81^ In cancer cells, the quiescence program of dormancy is induced by p38/MAPK activation,^81^ extracellular signal‐regulated kinase (ERK)^101^ and phosphoinositide 3‐kinase (PI3K)/protein kinase B (AKT) inactivation,^102^ as well as by decreasing glycolysis^103^ and glucose uptake.^104^, ^105^ A high ratio of p38/ERK induces cells to enter dormancy, whereas a high ratio of ERK/p38 induces dormant cells to grow and proliferate.^5^, ^101^, ^106^ Moreover, p38 signalling regulates stress associated protein promoting survival and resistance of dormant cells. p38 up‐regulate endoplasmic reticulum (ER) chaperone BiP and the activation of ER stress‐activated eukaryotic translation initiator factor 2alpha kinase RNA‐dependent protein kinase‐like ER kinase protect dormant cancer cells from chemotherapy‐induced stress.^107^ Targeting the p38/MAPK signalling pathway may be a therapeutic strategy to inhibit drug resistance mediated by dormancy.

### 3.4 Cell autophagy and dormancy of cancer cells

Autophagy indirectly regulates the cell cycle and participates in modulating the dormancy of cancer cells.^108^, ^109^ Inactivation of PI3K/AKT/mammalian target of rapamycin (mTOR) pathway promotes autophagy.^110^ 3‐Hydroxy butyrate dehydrogenase 2 overexpression induced autophagy, regulating intracellular ROS levels to inhibit the PI3K/AKT/mTOR pathway, thereby inhibiting growth of gastric cancer.^111^ Aplasia Ras homolog member I induce autophagy via inhibiting mTOR and PI3K signalling, up‐regulating ATG4 (autophagy protein), contributing to survival of dormant ovarian tumour cells.^112^ Down‐regulation of polo‐like kinase 4 (PLK4) induces autophagy to maintain tumour cell dormancy. In PLK4 knockdown colorectal cancer cells, the expression of autophagy‐related molecules: autophagy protein 5, beclin1 and microtubule‐associated protein light chain 3B (LC3B‐II) drastically are up‐regulated. The mRNA expression of dormancy signalling molecules, including leucine‐rich α−2 glycoprotein 1, p15, p27, BMP7 and p16 were up‐regulated. the expression of CDK4, CDK6 and cell proliferation markers such as proliferating cell nuclear antigen (PCNA) and Ki67 were down‐regulated.^113^ Autophagy promotes dormancy of cancer cells and protects them from apoptosis under environmental stress.

### 3.5 ER stress signalling pathway regulates the dormancy of cancer cells

ER stress signalling maintains a stable quiescent‐like phenotype, ensuring cell survival under chronic ER stress. Slow‐cycling/dormant cancer cells (SCCs) adapt to ER stress under hypoxic and low‐glucose conditions.^114^ ER stress facilitates the unfolded protein response to control the survival, growth arrest and death of stressed cells, which depends on the duration and severity of stress.^115^, ^116^, ^117^ Regulator of G protein signalling 2 (RGS2) expression allows SCCs to increase their survival ability in ER stress‐induced apoptosis by degradation of activating transcription factor 4 and eukaryotic initiation factor 2 phosphorylation. RGS2 negatively regulates the expression of genes encoding cell proliferation factors (such as Ki67, cyclin D1, CDK4, PCNA and CDK2) and positively regulates the expression of genes encoding CDK inhibitors to maintain cell cycle arrest in dormant cancer cells.^118^

### 3.6 Cdc42 GTPase regulate the dormancy of cancer cells

**FIGURE 1:**
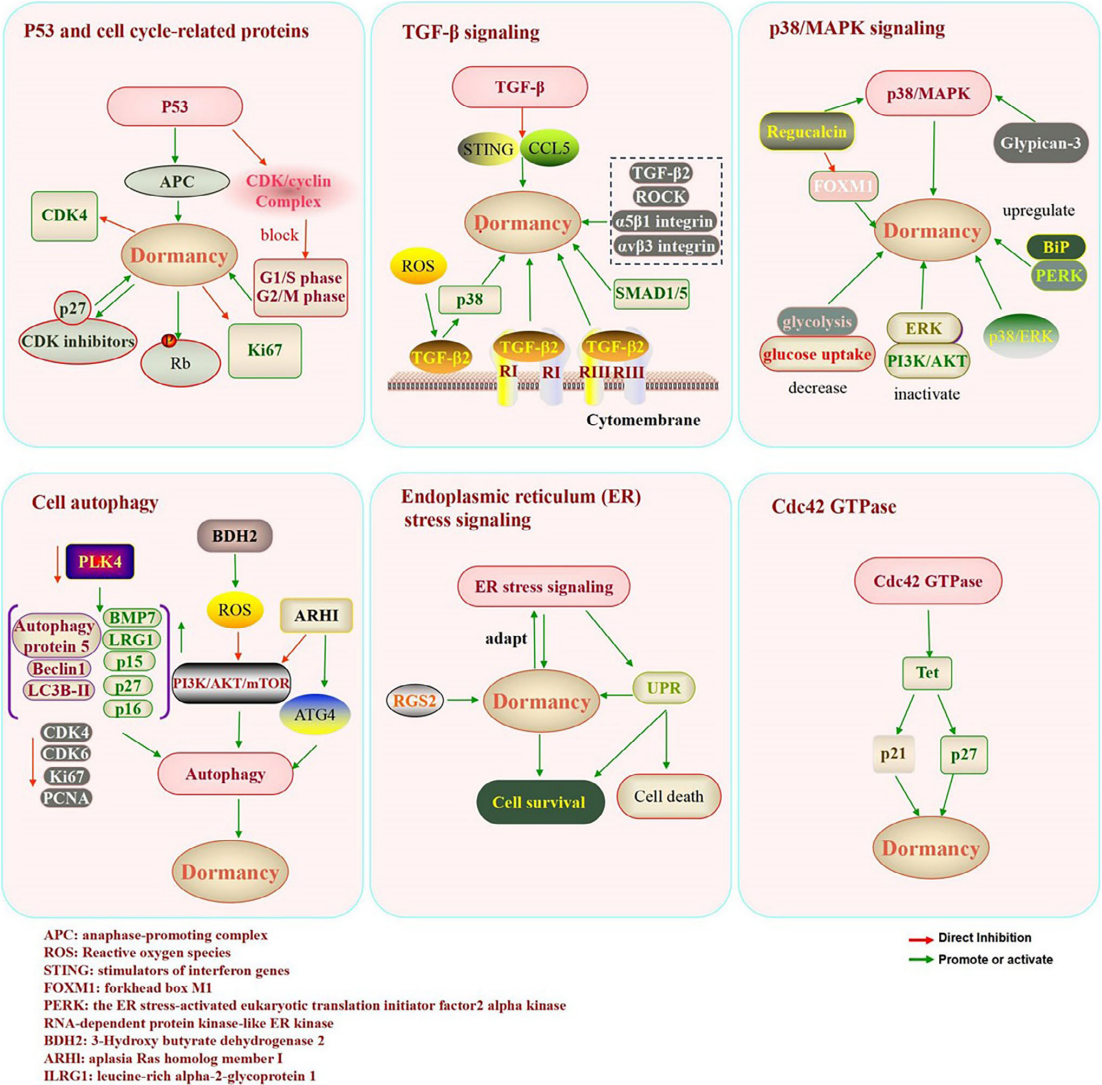
Molecular regulatory mechanism of dormant cancer cells. Regulatory mechanisms in dormant cancer cells can be divided into two categories: intracellular and extracellular. The dormancy of cancer cells is regulated by different molecules and signalling pathways, mainly involving: P53 and cell cycle‐related proteins, TGF‐β signalling pathway, p38/MAPK signalling pathway, cell autophagy, endoplasmic reticulum (ER) stress signalling and Cdc42 GTPase cycle.

The GTPase cell division cycle 42 (Cdc42) play important roles in the regulation of cancer cell dormancy. Increased ECM stiffness leads to Cdc42 translocation to the nucleus, promoting hydroxymethylating enzyme Tet2 expression, leading to hydroxymethylation of p21 and p27, and stimulating melanoma cells into dormancy.^119^ Cdc42 takes an action on regulating cell division and migration. The up‐regulation of Cdc42 may promote dormancy of prostate cancer cells^120^ (Figure 1).

## 4 PGCCS HAVE THE PROPERTIES OF DORMANT CANCER CELLS

PGCCs possess two major characteristics of dormant cancer cells: cell cycle arrest, and high migration and invasion capabilities. PGCCs deviate from the typical G1‐S‐G2‐M cell cycle. (Table 1 shows the different terms and explanations related to cell cycle arrest in PGCCs).^121^ Several studies have reported cell cycle arrest in PGCCs.^122^, ^123^, ^124^, ^125^, ^126^ In current reports, terminologies related to cell cycle arrest of PGCCs include G2 cell cycle arrest, G^Alert^/spG0/G0 cells, arrested in the G0/G1 phase, quiescence, slow‐cycling states, dormant state and proliferative arrest. Alhaddad L et al. discussed the radioresistance related to the proportion of dormant PGCCs by the Ki‐67/EdU staining features enriching in three subpopulations (G^Alert^, spG0, G0). In the three subpopulations, G^Alert^ cells are in a quiescence‐like state with moderate Ki‐67 expression, indicating that they were ready to re‐enter the cell cycle. The spG0 cells remain in a quiescent state, but with low level of Ki‐67 expression, indicating that they were in a deeper state of dormancy. G0 cells without Ki‐67 expression are fully quiescent. The G^Alert^, spG0, G0 states in quiescence‐ and/or senescence‐like may be an adaptive response of PGCCs to radiation stress.^127^, ^128^

**TABLE 1:** Terminologies and explanations of their reference cell cycle arrest of PGCCs.

Terminologies relate to cell cycle arrest of PGCCs	Explanation	References
Cell cycle arrest (Quiescence)	Cancer cell dormancy is a process whereby cells enter reversible cell cycle arrest (including G0/G1, G0, G1, G2 and G2/M arrest), termed quiescence.	^58^
Growth arrest	Cancer cell dormancy typically presents as growth arrest while retaining proliferative capacity and can be induced or reversed by a wide array of cell‐intrinsic and cell‐extrinsic factors.	^40^
Slow‐cycling	SCCs are a subpopulation of cancer cells that are relatively quiescent and exist in the G0/G1 cell‐cycle phase. SCCs are considered dormant cancer cells.	^129^
Non‐proliferation	Cellular dormancy refers to restricting proliferation at the cell level.	^130^
Non‐dividing	Most metastatic lesions are micro metastases that have the capacity to remain in a non‐dividing state called ‘dormancy’ for months or even years.	^131^
Dormant state	Dormant state is a relatively broad concept that includes various dormant cells	^131^, ^132^, ^133^

Abbreviation: SCCs, slow‐cycling cells.

Mallin et al.^12^ showed that PACC (PGCCs) exhibit increased motility and deformability mediated by increased mesenchymal‐related protein expression, and that PACC show increased metastatic potential. PGCCs have thicker and longer actin fibres with an abnormal overexpression of actin components compared with the diploid cancer cells, which confers PGCCs a slow yet persistent migratory phenotype. The actin network and dysregulated RhoA–ROCK1 pathway are potential mechanisms for PGCCs cytoplasmic biophysical changes.^134^ A recent study reported that up‐regulation of Cdc42 and p21‐regulated kinase 1 down‐regulates stathmin and increases the protein level of phosphorylated stathmin. Phosphorylated stathmin, existed in the nucleus of PGCCs and daughter cells, promotes microtubule aggregation and regulates cytoskeletal remodelling. Cdc42 overexpression takes a part in the invasion and migration of PGCCs and daughter cells.^123^ In summary, PGCCs maintain cell cycle arrest and exhibit high migration and invasion rates when dormant.

### 4.1 PGCCs‐related signalling pathways are consistent with the molecular characteristics of dormant cells

A previous proteomic analysis indicates that PGCCs are associated with dormancy. (1) Alteration in several cell cycle‐related proteins (FOXM1, cyclin E, cyclin D1, CDK6, checkpoint kinase 1 (CHK1), cyclin A2, Checkpoint kinase 2 (CHK2), S100 calcium‐binding protein A10 (S100A10), S100A6 and S100A4) may be involved in the formation of PGCCs. (2) Up‐regulation of proteins (Heat Shock Protein Family A Member 1B, Heat Shock Protein Family A Member 1A and annexin A1) protects cells from stress, whereas down‐regulation of stress‐induced phosphoprotein 1 inhibits proliferation. (3) Cell motility‐related proteins (cathepsin D, cathepsin B, vimentin, high mobility group box protein 1 (HMGB1), annexin A2, integrin β2 and Ezrin) were increased in PGCCs and their daughter cells. (4) PGCCs may undergo different metabolic processes (up‐regulation of lactate dehydrogenase A and enolase 1) and are more adaptable to stress than diploid cells.^135^ In summary, PGCCs share molecular characteristics and regulatory mechanisms with dormant cancer cells, including cell cycle arrest, high migratory ability and adaptation to adverse microenvironments.

### 4.2 PGCCs achieve dormancy through endoreplicative cell cycle

Fluorescence imaging revealed that PGCCs undergo endoreplicative cycles with alternating S and G phases, accumulating DNA and enlarging the cell/nucleus without dividing.^22^ Endoreduplication is a cell‐cycle variation in which the nuclear genome replicates without mitosis, resulting in polyploidy.^136^ Abnormal karyotypes and polyploid programs of PGCCs are attributed to endoreplication.^26^, ^137^ Aurora kinase A/B (AURKA/B), which abnormally regulates cytokinesis in PGCCs, accounts for the functional differences between normal mitosis and endoreplication in PGCCs.^138^, ^139^, ^140^ Endoreduplication and polyploidisation are survival strategies adopted by dormant PGCCs to adapt to harsh environmental conditions. The cell cycle of PGCCs can be classified into four stages. (1) *Initiation*: Multiple stressors can induce endoreplicative cell cycles in cancer cells, leading to the formation of PGCCs for survival after anti‐mitotic treatments.^139^, ^141^ When treated with cisplatin, carboplatin or paclitaxel, the number of PGCCs (>4N) increases rapidly, while the number of diploid (2N) cancer cells decreases through apoptosis.^26^, ^142^, ^143^ (2) *Self‐renewal*: Polyploid cells enter the endoreplication cell cycle via the endomitosis or endocycle, which allows tumour cells to grow in a polyploid state.^139^, ^141^ (3) *Termination*: PGCCs generate diploid cells via budding, fragmentation, fission and cytofission. PGCCs generate diploid progeny, which may be related to changes in AURKA/B.^139^, ^140^ (4) *Stability*: Diploid daughter cells have acquired new genomes and resume mitosis.^139^, ^141^ Study of Richards et al.^140^ indicated that the explosive growth of PGCCs at day 21 coincided with a transition from slow to rapid growth in xenograft tumours. The initiation and self‐renewal stages of PGCCs are similar to the cell cycle arrest of dormant cancer cells, whereas the termination and stability stages resemble the reactivation stage.

**FIGURE 2:**
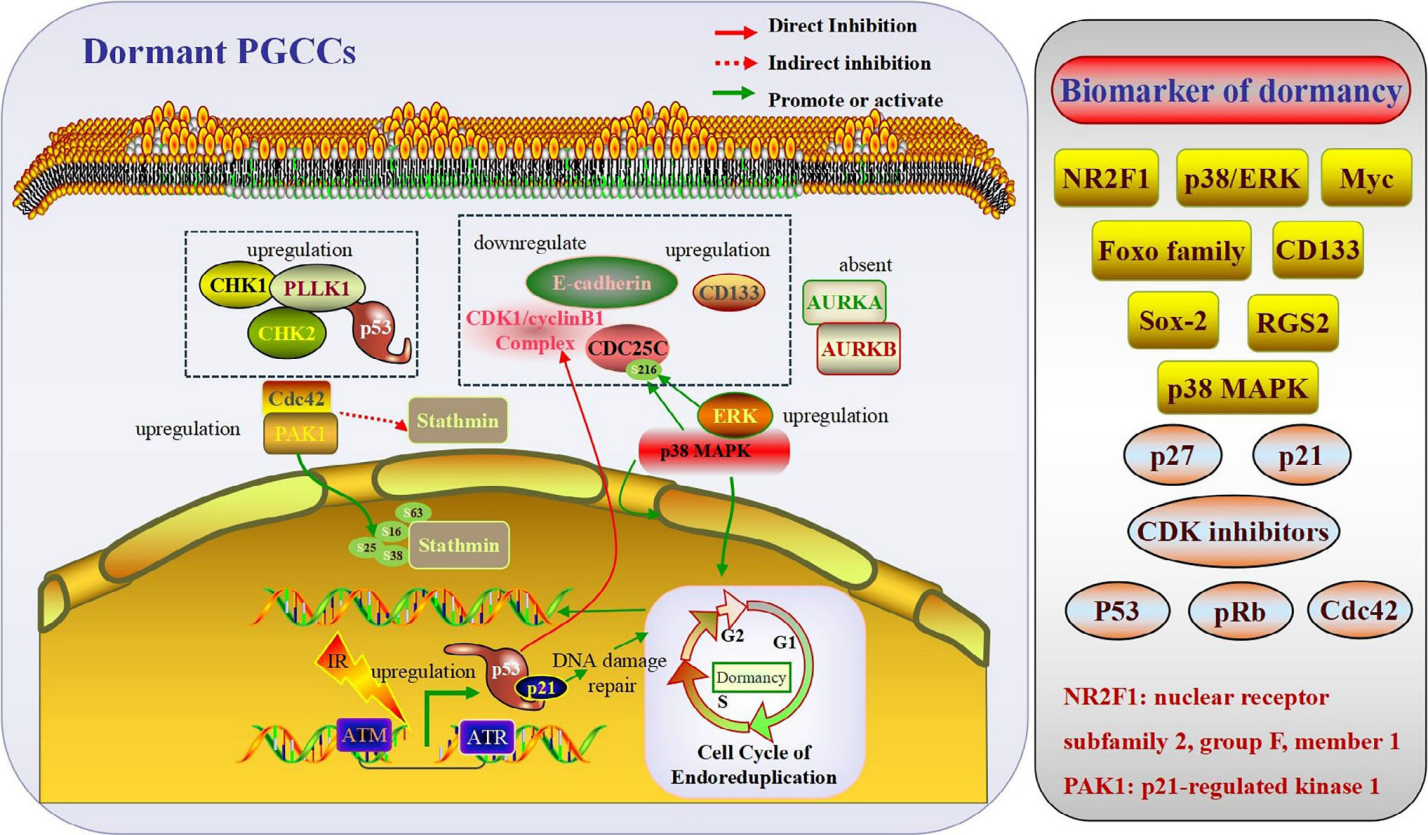
Molecular regulatory mechanisms of dormant PGCCs. Cancer cells enter a dormant state and resist various stressors by maintaining a cell cycle arrest. PGCCs possess major characteristics of dormant cancer cells, including cell cycle arrest. PGCCs represent a dormant form of the stress‐induced cancer cell cycle. PGCCs‐related signalling pathways were consistent with the molecular characteristics of dormant cells. Alterations in several cell cycle‐related proteins (PLK1, AURKA/B, P53, CHK1, CHK2, Cdc42, PAK1, Stathmin, p21, ATM, ATR, p38MAPK, ERK, CDK1, cyclinB1 and CD133) may maintain PGCCs dormancy.

The disruption of cell cycle checkpoints mediated by CDK is necessary for the endoreplication of PGCCs. The p16/pRb and P53/p21 pathways can regulate the expression of CDK. Severe DNA damage from chemotherapy or hypoxia induces P53 up‐regulation and cells with mutant P53 become aneuploid.^127^ Endoreduplication increases the cell size, DNA content and nuclear heterogeneity in response to various stressors.^144^, ^145^ In PGCCs, CDK1 and cyclin B1 expression is down‐regulated, whereas the expression of P53 is up‐regulated.^124^, ^126^, ^146^ P53 drives cells into a safer and more favourable dormant state, enhancing the adaptability and survival of PGCCs to irradiation stress.^66^, ^147^ The CHK1, CHK2–pCDC25C‐Ser216–cyclin B1–CDK1 and AURKA–Polo‐like kinase 1 (PLK1)–pCDC25C‐Ser198–cyclin B1–CDK1 signalling pathways are participant of PGCCs formation.^25^, ^126^ ataxia telangiectasia‐mutated gene (ATM)/ataxia telangiectasia mutated and Rad3‐related (ATR)‐P53‐p21 axis induces cell dormancy and cell cycle arrest.^147^ In addition, the subcellular localisation of cell cycle‐related proteins (cyclin B1, CDC25C, CDK1) is also correlated with the formation of PGCCs.^146^ These proteins play crucial roles in maintaining PGCCs dormancy and in restoring cell proliferation (Figure 2).

### 4.3 The endoreplicative feature endows PGCCs drug resistance

Under stress, cells respond in various ways to maintain balance.^139^, ^141^ Polyploidy can also be used to achieve greater functionality and independence. Polyploidy exhibit enhanced stress resistance and resist chemotherapeutic drugs (doxorubicin, vincristine, paclitaxel, etc.) at extreme doses.^18^, ^121^, ^134^, ^138^, ^139^, ^148^, ^149^ PGCCs survive at high docetaxel concentrations and may undergo EMT to survive in regions with high drug concentrations.^150^ Nasopharyngeal cancer (NPC) is an Epstein–Barr Virus (EBV)‐related squamous cell carcinoma of the head and neck. EBV can promote EMT in dormant PGCCs to enhance stemness.^21^ The dormancy of PGCCs can be reactivated by specific signals, causing late‐stage relapse of NPC and other malignancies.^23^ Continuous staurosporine treatment induces PGCCs formation and maintains quiescence in A549 cells for several months.^151^

In addition, polyploidy allows short‐term metabolic changes to cope with oxidative stress.^152^ Once cancer cells obtain this ability, they also have the ability to survive and respond to stress in tumour microenvironment (TME).^17^, ^150^, ^153^ After paclitaxel causes mitotic crisis and cancer cell death, the remnant cells undergo genomic shock, activating emergency survival mechanisms.^154^ Dormant cancer cells allow for energy conservation and distribution under poor environmental conditions.^155^ PGCCs are rich in mitochondria, which support the survival of cancer cells by producing ATP. Adibi et al.^156^ reported increased lipid droplet formation has been observed in PGCCs. Lipids likely serve as energy stores that help PGCCs endure stress.^157^ The increased lipid droplets may serve as energy storage reservoirs that enable cancer cells to effectively endure long‐term stress conditions. In PGCCs‐enriched samples, glucose‐1‐phosphate and glutamine were significantly differentially abundant.^158^ Increasing the volume of DNA without division during dormancy is advantageous when energy is limited or growth is rapid, allowing PGCCs to reserve more energy (lipids, proteins and carbohydrates) and survive extended periods of dormancy.^159^, ^160^ It also protects against toxins and oxidative stress by increasing the expression of RNA and proteins involved in protection pathways.^17^ Third, polyploidy allows cell‐cycle arrest and genetic modifications with whole‐genome doubling.^17^

## 5 REACTIVATION OF DORMANT PGCCs PROMOTES TUMOUR INVASION AND METASTASIS

Dormant PGCCs can be activated by external signals such as growth factors and cytokines. These signals promote cell proliferation, causing dormant tumour cells to re‐enter the cell cycle and initiate metastasis.^65^

### 5.1 PGCCs exit dormancy and produce daughter cells via asymmetric division

After treatment, PGCCs require a latency period (dormancy) to restore viability and produce daughter cells.^18^ The length of the dormant period depends on the chemotherapy dose and radiotherapy intensity.^11^, ^125^ PGCCs can exit dormancy and resume proliferation after restoring favourable conditions. Gilbertson et al.^137^ described the manner of PGCCs producing progeny cells as ‘neosis’. Asymmetric division leads to the loss of genetic material in the mononuclear daughter cells, thereby increasing their genetic instability and heterogeneity.^10^ Extracellular HMGB1 enhances the asymmetric division of PGCCs during tumour regrowth. Moreover, overexpression of the receptor for advanced glycation end products (RAGE), p‐p38 and p‐ERK in PGCCs suggests the involvement of HMGB1/RAGE/p38 and ERK pathways in asymmetric division of PGCCs.^19^, ^121^, ^159^ In addition, S‐phase kinase‐associated protein 2, stathmin, cyclin E, CDK2, cyclin B1, p‐AKT, phosphoglycerate kinase 1, protein kinase C, p38 and MAPK are also up‐regulated in daughter cells produced by PGCCs via asymmetric division.^161^

Acid ceramidase (ASAH1) is a lysosomal enzyme that plays an essential role in the survival of PGCCs by hydrolysing ceramide to sphingosine. This enables the PGCCs to produce progeny via lysosome‐mediated cell division.^137^ High levels of C16‐ceramide inhibit PGCCs progeny formation. However, this inhibition can be prevented by inhibiting ASAH1 or by expressing functional P53. In contrast, P53 loss and low C16 ceramide levels, due to altered sphingolipid metabolism, promote PGCCs progeny formation.^162^

### 5.2 PGCCs‐derived daughter cells have strong abilities of proliferation, migration and invasion

**FIGURE 3:**
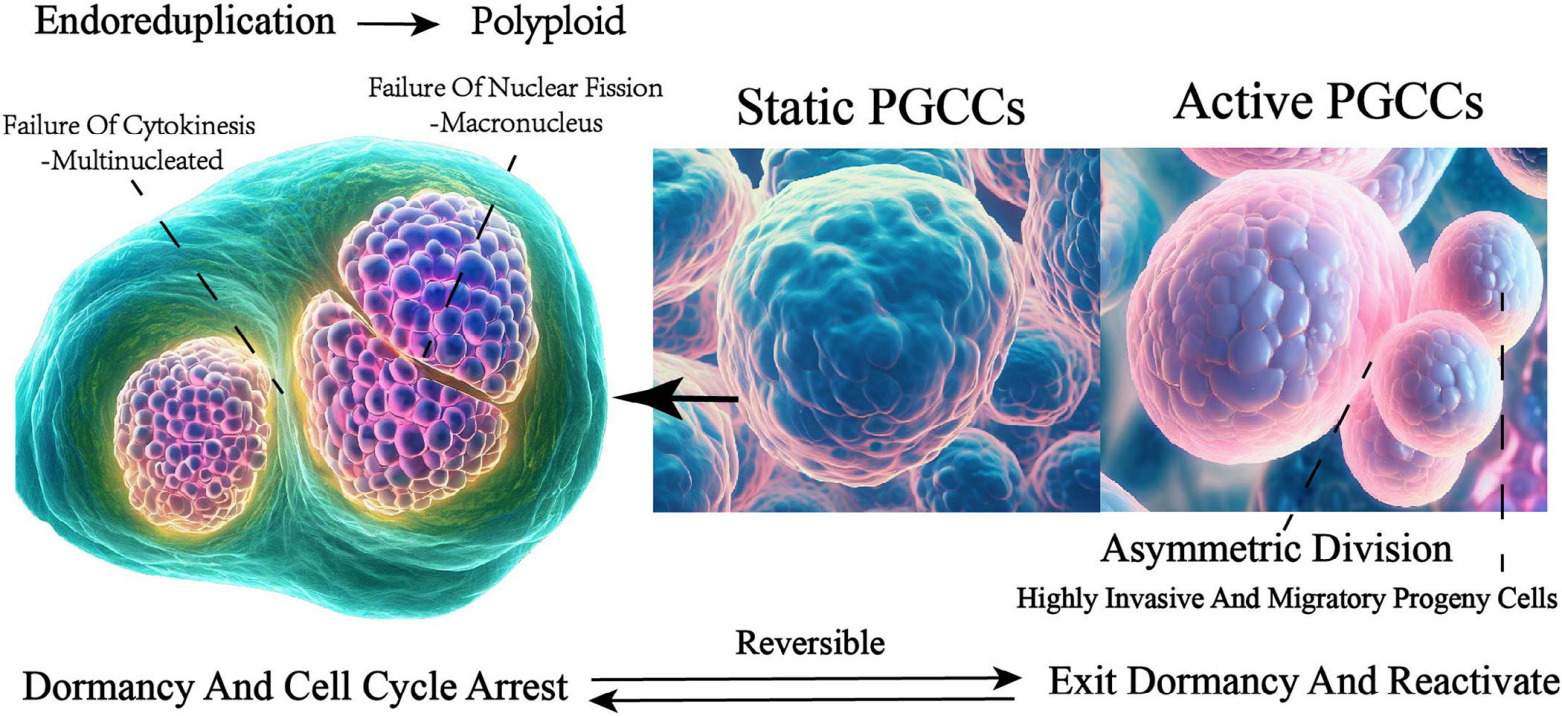
PGCCs dormancy and activation pattern diagram. PGCCs achieve dormancy via an endoreplicative cell cycle. Endoreduplication is a cell‐cycle variation in which the nuclear genome replicates without mitosis, resulting in polyploidy. The abnormal karyotype and polyploidy of PGCCs have been attributed to endoreplication. Polyploid cells continue their endoreplicative cell cycle through endocycles or endomitosis, generating mono‐ and multi‐nucleated PGCCs. PGCCs retain their proliferative potential during dormancy and stimulate tumour cell proliferation through asymmetric division. Active PGCCs, dormant PGCCs is reactivated and re‐enter cell cycle via asymmetric cell division.

The highly invasive and migratory characteristics of PGCCs‐derived daughter cells have been demonstrated in many studies.^24^, ^123^, ^134^, ^149^, ^163^, ^164^, ^165^, ^166^ PGCCs‐derived daughter cells can easily metastasize to the lymph nodes or distant organs by expressing EMT‐related proteins (Snail, Slug and Twist).^167^ Ezrin, S100A4 and 14‐3‐3ζ/δ are involved in the migration and invasion of PGCCs‐derived daughter cells via cytoskeletal constructions.^165^ The nuclear localisation of S100A10 modified by SUMOylation is associated with the high proliferation and migration of PGCCs‐derived daughter cells by regulating the expression of neutrophil defensin 3, receptor‐type tyrosine‐protein phosphatase N2 and rho guanine nucleotide exchange factor 18.^164^ PGCCs‐derived daughter cells express high levels of vimentin which enhances the migration and invasion.^24^ P62‐dependent SUMOylation of vimentin locates in the nuclear and acts as a transcription factor that regulates Cdc42, cathepsin B and cathepsin D to promote PGCCs‐derived daughter cells invasion and migration^168^ (Figure 3).

### 5.3 Cross‐talk between cytokines and PGCCs

**FIGURE 4:**
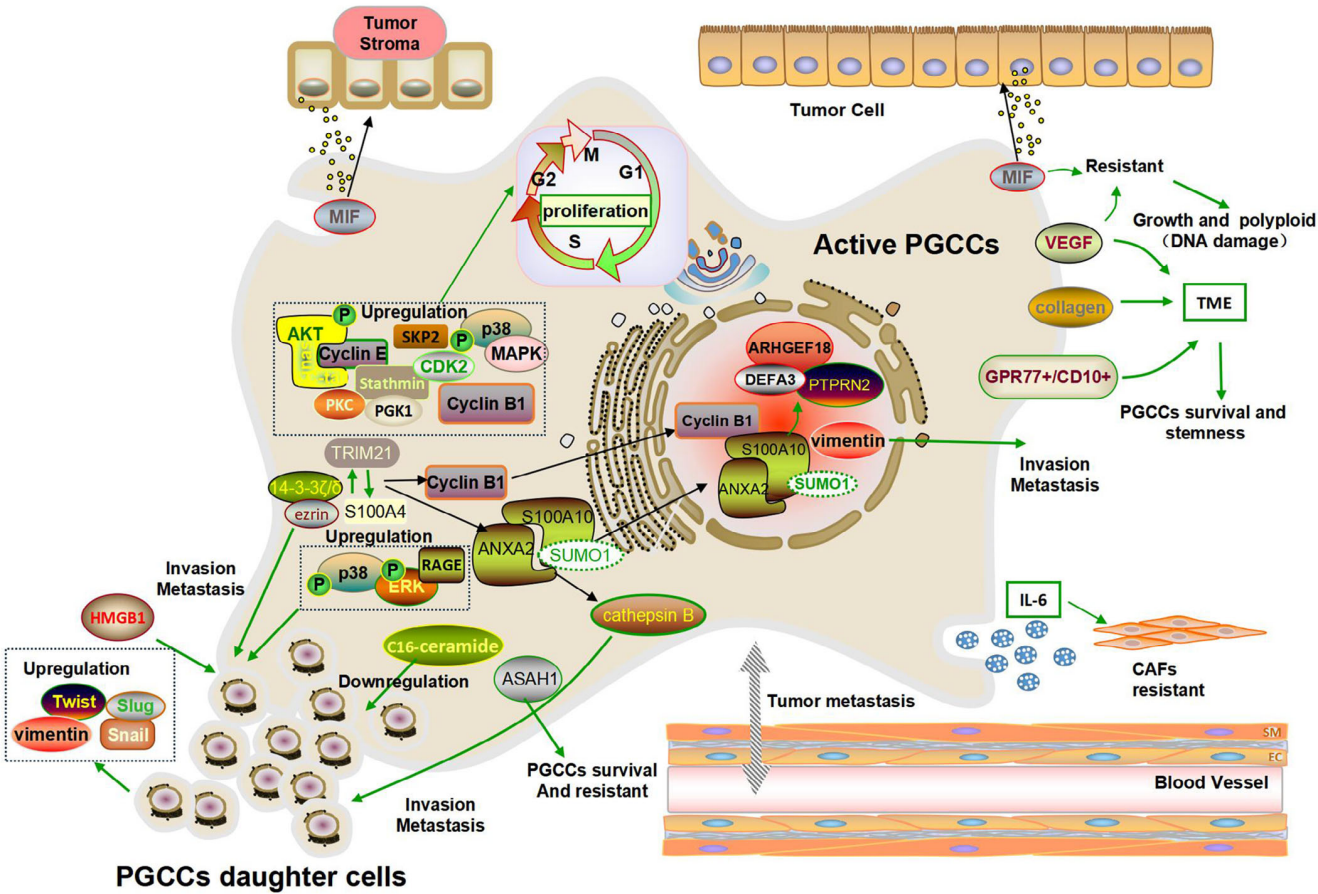
Reactivation of dormant PGCCs via asymmetric division promotes tumour invasion and metastasis. PGCCs retain their proliferative potential during dormancy and stimulate tumour cell proliferation through asymmetric division to produce daughter cells. PGCCs can exit dormancy and recur, thereby generating aggressive diploid progeny. Daughter cells exhibited EMT features as well as high invasion and migration capabilities. Tumour microenvironment (TME) is a dynamic network of diploid cells, PGCCs, daughter cells, stromal cells, blood vessels and vasculogenic mimics. PGCCs can be activated by external signals such as growth factors and cytokines. These signals promote cell proliferation, causing dormant PGCCs to re‐enter the cell cycle and initiate metastasis.

Crosstalk between cytokines and PGCCs plays a critical role in cancer progression. Growth factors and cytokines derived from PGCCs, including macrophage migration inhibitory factors (MIF) and VEGF, can promote resistance to chemotherapy.^169^, ^170^ MIF derived from PGCCs may allow cancer cells to grow and proliferate. Ahmad et al.^171^ reported a relationship between high‐risk human cytomegalovirus (HCMV) strains, PGCCs and cytokines in breast cancer. Both TGF‐β and IL‐6 can activate signalling pathways in EMT and drive cancer progression. HCMV may utilise cytokines like IL‐10 and TGF‐β to create a tumour‐supportive microenvironment and modulate pathways controlling PGCCs formation and their stem‐like properties.^171^ In addition, paclitaxel treatment can induce an ‘inflammatory storm’ dominated by IL‐6 production during PGCCs formation. IL‐6 acts in an autocrine manner to facilitate the acquisition of stemness properties and survival of PGCCs. The PGCCs‐derived IL‐6 stimulates fibroblasts to increase collagen production, enrichment of the GPR77+/CD10+ cancer‐associated fibroblasts (CAFs) subpopulation and VEGF expression.^26^, ^172^ PGCCs also utilise IL‐6 as a paracrine molecule to reprogram normal fibroblasts into CAFs. Reprogrammed CAFs maintained PGCCs stemness, promoted angiogenesis and metastasis and modified the TME to favour the survival of PGCCs after chemotherapeutic drug treatment.^172^ PGCCs can increase the cell‐cell interactions with surrounding cancer cells. They express high levels of surface markers such as N‐cadherin, and OB‐cadherins, as well as integrin alpha 5 and fibronectin, which likely contribute to their interactions with other cells in the TME^173^ (Figure 4 and Table 2).

**TABLE 2:** Main similarities and differences between dormant PGCCs and cancer cells.

Feature	Dormant PGCCs	Dormant cancer cells
**Same point**		
Origin	Response to stress, including chemotherapy, ionising radiation and hypoxia^170^	Due to hypoxia, nutrient deprivation, immune surveillance and chemotherapy^82^
Cell cycle status	Static^21^, ^22^, ^23^, ^24^, ^174^	Cell cycle arrest in the G0 or G1 phase^175^
	Proliferative arrest^25^	Growth arrest^58^
	Arrested in the G0/G1 phase^26^	
Drug resistance	Prevent the toxic effects of systemic therapy^19^	Driving treatment resistance^118^
Proliferation	Asymmetric division (neosis)^15^	Reactivate to promote cell proliferation^175^
Recurrence and metastasis	Driving recurrence and promote metastasis^162^	Re‐enter the cell cycle and initiate the metastasis^65^
Microenvironmental signals	Adaptable to the harsh microenvironments^135^	Adaptation to tumour microenvironment of tumour hypoxia and nutrient limitation^176^
**Unique characteristics of PGCCs**		
Stemness and differentiation	Characteristics of stem cells, such as self‐renewal and differentiation ability^18^, ^157^	
	Expression of the embryonic stem cell markers OCT4, NANOG, SOX2 and SSEA1^138^	
	Can differentiate into adipose, cartilage and bone^18^	
	Express embryonic haemoglobin with strong oxygen binding ability, promoting survival of tumour cells in a hypoxic microenvironment^20^	
Nuclear heterogeneity	PGCCs have single giant or multiple nuclei. Multinuclear PGCCs are more likely to be produced when nuclear replication and separation are completed, and cytoplasmic separation fails. Single giant nuclear PGCCs would appear if both nuclear and cytoplasmic separation fails at the same time.^15^, ^16^, ^17^	
EMT	Daughter cells derived from PGCCs undergo EMT phenotype changes^149^, ^177^	
Cell division mode	Asymmetric division^135^	

## 6 POTENTIAL THERAPEUTIC STRATEGY FOR TARGETING PGCCs

In clinical practice, most chemotherapeutic drugs are designed to disrupt mitosis and rapidly divide the cancer cells. Common antimitotic drugs such as aclitaxel, docetaxel, vinorelbine, vinblastine, the PLK1 inhibitor BI‐6727 (Volasertib), CENPE inhibitor (GSK923295), EG5 inhibitors (Ispinesib) and VS‐83, inhibit tumour cell growth and division by interfering with mitosis.^178^ However, PGCCs is resistant to antimitotic drugs. Moreover, these drugs can damage normal tissues, leading to nephrotoxicity, bone marrow suppression and peripheral neuropathy.^179^ Currently, drugs targeting PGCCs are still in preclinical stages. The development of drugs targeting PGCCs may be an important strategy for reducing the occurrence of drug‐resistant progeny. Small molecular chemical reagents targeting PGCCs including: (1) reduced the number of PGCCs (mTOR inhibitor and poly ADP ribose polymerase [PARP] inhibitor, hydroxychloroquine, nelfinavir, rapamycin, paclitaxel and ganciclovir). In tumours with cyclin E1 amplification, PGCCs highly expressed CHK2 and co‐stained with the DNA damage marker, gamma histone H2AX, indicating that these proteins take part in maintaining the survival of PGCCs. Dual treatment with an mTOR inhibitor and a PARP inhibitor significantly reduced the number of PGCCs.^180^ Two autophagy inhibitors, hydroxychloroquine and nelfinavir, reduced the number of progeny generated from PGCCs in high‐grade serous carcinoma (HGSC), thereby inhibiting their growth and proliferation, which prevents PGCCs from repopulating tumours. Rapamycin also prevented PGCCs colony outgrowth. Clinical trials using hydroxychloroquine, nelfinavir and/or rapamycin after chemotherapy may be important for targeting PGCCs.^181^ Attempts to reduce the number and proliferation of PGCCs and limit their formation using paclitaxel and ganciclovir have shown some effectiveness. However, the efficacy of the drug combinations may vary depending on the cell line used.^157^ (2) Prevented formation of PGCCs (tocilizumab, apigenin, mifepristone and PARP inhibitors, BML‐275, PRL3‐zumab). Tocilizumab and Apigenin block IL‐6 and reduce PGCCs formation, thereby inhibiting tumour growth.^172^ Zhang et al.^22^ targeted PGCCs in ovarian cancer and found that mifepristone could synergistically work with PARP inhibitors to promote apoptosis of cells undergoing endoreplication, thereby blocking PGCCs formation and enhancing the therapeutic effect of PARP inhibitors. Chemotherapy can induce nasopharyngeal carcinoma cells to form dormant PGCCs. Use of the autophagy inhibitor BML‐275, an AMP‐activated protein kinase inhibitor, before chemotherapy can prevent PGCCs formation and reduce nasopharyngeal carcinoma relapse and metastasis.^23^ Overexpression of phosphatase in regenerating liver 3 (PRL3) in PGCCs causes abnormal cell division. PRL3^+^ PGCCs can tolerate the genotoxic stress induced by chemotherapy by inhibiting the ATM DNA damage signalling pathway, which increases their survival and drug resistance. PRL3‐zumab is a humanised antibody that targets the PRL3 oncogene and reduces tumour metastasis and recurrence by targeting PRL3^+^ PGCCs. PRL3‐zumab is considered a potential ‘adjuvant immunotherapy’ that can be used after tumour removal surgery to eliminate PRL3^+^ PGCCs and prevent tumour metastasis and recurrence.^182^ IL‐1β is involved in regulating the resistance of docetaxel chemotherapy, which is achieved by regulating the formation of PGCCs. Thus, treatments targeting IL‐1β may help improve the efficacy of chemotherapy drugs by reducing the formation of PGCCs.^122^ (3) Reduced lipid droplets and cholesterol levels in PGCCs. Zoledronic acid (ZA) is used to treat various cancers, including breast, bladder, renal and lung. PGCCs undergo changes in lipid metabolism, thereby increasing lipid droplets and cholesterol levels. Lipids serve as energy storage agents, enabling cancer cells to endure long‐term stressful conditions. ZA significantly reduced lipid droplets and cholesterol levels in PGCCs. This indicates that targeting lipid metabolism in PGCCs may represent a promising strategy for overcoming chemoresistance and improving the success rate of cancer treatment.^156^ (4) Induced‐differentiation. PGCCs cultured in medium containing 3‐isobutyl‐1‐methylxanthine, insulin, dexamethasone and rosiglitazone can be induced to transdifferentiate into mature functional adipocytes. Ishay‐Ronen et al.^183^ reported that invasive and metastatic breast cancer cells can be differentiated into adipocytes by MEK inhibitors and rosiglitazone, and invasion and metastasis abilities were inhibited in breast cancer (Tables 3 and 4).

**TABLE 3:** Published literature about PGCCs across different cancer types.

Cancer type	Cell lines	Chemical reagents	The roles of PGCCs in cancer	References
Ovarian cancer	High‐grade serous carcinoma and MDA‐HGSC‐1 (patient source), HEY, SKOv3, OVCAR3, CAOV3, IGROV1, OVCA‐432, OVCAR8, OVCAR5, PEO‐1, A2780, SCOV‐3, NIHOVCAR3, COV318, OVCAR4	Paclitaxel, cobalt chloride, triptolide, HA15 (Selleckchem, Zurich, Switzerland), thapsigargin (Enzo LifeSciences, Lausen, Switzerland), tunicamycin (Enzo LifeSciences), carboplatin, docetaxel, olaparib, cisplatin, mTOR inhibitor	Associated with poor prognosis Chemoresistance and cancer stem cell characteristics Promote invasion and migration. Contributes to the evolution and remodelling of cancer Associated with the formation of vasculogenic mimics	^18^, ^22^, ^25^, ^126^, ^138^, ^139^, ^161^, ^166^, ^172^, ^180^, ^181^, ^184^, ^185^, ^186^, ^187^, ^188^
Breast cancer	MCF‐7, MDA‐MB‐231, MDA‐MB‐453, SKBR‐3, BT‐549, 4T1, HMECs	Cisplatin, docetaxel, cobalt chloride, triptolide, irradiation, human cytomegalovirus, paclitaxel, olaparib	Chemoresistance Promote invasion, metastatic dissemination and migration Cancer stem cells characteristics Associated with the malignant grade of breast tumour	^10^, ^18^, ^19^, ^24^, ^25^, ^126^, ^149^, ^156^, ^157^, ^158^, ^171^, ^189^, ^190^, ^191^
Colorectal cancer	LoVo, HCT116, Caco‐2	Cobalt chloride, arsenic trioxide, capecitabine, oxaliplatin, irinotecan, irradiation	Daughter cells derived from PGCCs have strong proliferation, invasion and migration abilities Contributed to expansion of a cell subpopulation with cancer stem cells characteristics Associated with the differentiation of colorectal cancer Associated with the formation of vasculogenic mimics Chemoresistance	^11^, ^20^, ^121^, ^164^, ^165^, ^168^, ^177^, ^192^, ^193^, ^194^, ^195^
Glioma	A172, C6, U251, Hs683	Cobalt chloride, paclitaxel, puromycin	Enhanced the polarisation of tumour‐associated macrophages into an M2 phenotype with relevance to immunosuppression and malignancy in GBM Associated with the formation of vasculogenic mimics, as well as the malignancy and prognosis of tumours Enhanced temozolomide resistance	^15^, ^166^, ^196^, ^197^
Lung cancer	A549, H1299, NSCLC, HBEC	Docetaxel, staurosporine, SB 328437, SB 297006 (CCR3 antagonists), aurora kinase inhibitors	Chemoresistance Enhanced migration and proliferation abilities Promote the recurrence	^122^, ^178^, ^193^
Prostatic cancer	PC3, PPC1	PRL3 overexpression induction, cisplatin, docetaxel, irradiated	Promote the recurrence and metastasis	^12^, ^137^, ^182^
Nasopharyngeal carcinoma	5–8F, CNE2, C666‐1	Paclitaxel	Correlate with the recurrence and metastases of NPC EBV promotes VM formation by PGCCs in vitro and in vivo which correlates with the growth of NPC cells	^21^, ^23^
Laryngeal cancer	TU212	Paclitaxe	Associated with poor prognosis of patients	^143^
Prostate cancer	Prostate epithelial cells infected by human cytomegalovirus	Human cytomegalovirus	PGCCs with the poor prognosis of prostate cancer	^198^
Leukaemia	K562	5‐Azacytidine	Resistant to chemotherapeutic agent and serum starvation stress	^159^
Oral cancer	Cal33, FaDu	Cisplatin	Chemoresistance	^199^

**TABLE 4:** Current/proposed therapies that may inhibit the formation of PGCCs.

Current/proposed therapies for PGCCs	Mechanisms	Advantages	Disadvantages	Citations
Combination therapies				
mTOR inhibitor and PARP inhibitor	Inhibiting the mTOR signalling pathway Blocking the activity of PARP enzymes	Reduced the number of PGCCs	Not reported	^180^
Mifepristone and PARP inhibitors	Mifepristone promotes apoptosis of cells undergoing endoreplication. PARP inhibitors target the DNA repair pathway in cancer cells	Blocking PGCCs formation and enhancing the therapeutic effect of PARP inhibitors		^22^
Paclitaxel and Ganciclovir	Inhibit tumour cell mitosis and induce cell apoptosis; inhibit virus growth and replication	Reduce the number and proliferation of PGCCs	The therapeutic efficacy may vary depending on different cell lines	^157^
Autophagy inhibitors				
Hydroxychloroquine, rapamycin, nelfinavir	Regulatory autophagy	Reduced the number of PGCCs	Not reported	^181^
BML‐275	AMP‐activated protein kinase inhibitor	Prevent PGCCs formation and reduce nasopharyngeal carcinoma relapse and metastasis		^23^
Induced differentiation				
3‐Isobutyl‐1‐methylxanthine + insulin+ dexamethasone+ rosiglitazone	PGCCs can be induced to transdifferentiate into mature functional adipocytes		Not reported	^124^, ^183^
Other therapies				
Tocilizumab, apigenin	Block interleukin‐6	Reduce PGCCs formation	Not reported	^172^
PRL3‐zumab	Targets the PRL3 oncogene	Eliminate PRL3^+^ PGCCs and prevent tumour metastasis and recurrence		^182^
Zoledronic acid	Induce apoptosis of osteoclasts	Reduced lipid droplets and cholesterol levels in PGCCs		^156^

## 7 CONCLUSION

PGCCs have emerged as key regulators of tumour dormancy and reactivation. Abnormal cell cycle regulation in PGCCs enables them to enter a reversibly dormant state and evade cancer therapy. Specifically, PGCCs achieve dormancy through endoreduplication cycles mediated by CDK inhibitors and tumour suppressors like P53. Polyploidy allows PGCCs to increase their DNA content and cell size, thereby enhancing their resistance to stress. Furthermore, PGCCs can exit dormancy and recur via asymmetric division, thereby generating aggressive diploid progeny. Daughter cells exhibit EMT features, as well as high invasion and migration capabilities. Therefore, understanding the mechanisms underlying PGCCs dormancy and reawakening holds great promise for the better management of tumour recurrence, metastasis and drug resistance. Further research is needed to fully elucidate the complex molecular events driving PGCCs behaviour. Developing clinical interventions targeting PGCCs survival and division may help to overcome cancer relapse after therapy .

## AUTHOR CONTRIBUTIONS

*Writing – original draft*: Yuqi Jiao. *Writing – original draft*: Yongjun Yu. *Writing – review and editing*: Minying Zheng. *Writing – review and editing*: Man Yan. *Writing – review and editing*: Jiangping Wang. *Conceptualisation and Supervision*: Yue Zhang. *Investigation, Writing – original draft, Writing – review & editing*: Shiwu Zhang. All authors certify that they have participated sufficiently in this work to take public responsibility for the content. All authors have read the journal's authorship agreement, reviewed and approved the manuscript.

## CONFLICT OF INTEREST STATEMENT

The authors declare no conflict of interest.

## ETHICS STATEMENT

Not Applicable.

## Data Availability

Not available.
